# Comparable Effects of the Androgen Derivatives Danazol, Oxymetholone and Nandrolone on Telomerase Activity in Human Primary Hematopoietic Cells from Patients with Dyskeratosis Congenita

**DOI:** 10.3390/ijms21197196

**Published:** 2020-09-29

**Authors:** Margherita Vieri, Martin Kirschner, Mareike Tometten, Anne Abels, Benjamin Rolles, Susanne Isfort, Jens Panse, Tim H. Brümmendorf, Fabian Beier

**Affiliations:** Department of Hematology, Oncology, Hemostaseology and Stem Cell Transplantation, Medical School, RWTH Aachen University, 52074 Aachen, Germany; mvieri@ukaachen.de (M.V.); mkirschner@ukaachen.de (M.K.); mtometten@ukaachen.de (M.T.); aabels@ukaachen.de (A.A.); brolles@ukaachen.de (B.R.); sisfort@ukaachen.de (S.I.); jpanse@ukaachen.de (J.P.); tbruemmendorf@ukaachen.de (T.H.B.)

**Keywords:** dyskeratosis congenita, androgen, danazol, telomerase, oxymetholone, nandrolone, telomeropathy, telomere

## Abstract

Dyskeratosis congenita (DKC) is a rare inherited disease of impaired telomere maintenance that progressively leads to multi-organ failure, including the bone marrow. By enhancing telomerase activity, androgen derivatives (ADs) are a potential therapeutic option able to re-elongate previously shortened telomeres. Danazol, oxymetholone, and nandrolone are ADs most frequently used to treat DKC. However, no direct in vitro analyses comparing the efficacy of these ADs have been conducted so far. We therefore treated mononuclear cells derived from peripheral blood and bone marrow of four patients with mutations in telomerase reverse transcriptase (*TERT*, *n* = 1),in the telomerase RNA component (TERC, *n* = 2) and in dyskerin pseudouridine synthase 1 (*DKC1*, *n* = 1) and found no substantial differences in the activity of these three agents in patients with *TERC/TERT* mutations. All AD studied produced comparable improvements of proliferation rates as well as degrees of telomere elongation. Increased *TERT* expression levels were shown with danazol and oxymetholone. The beneficial effects of all ADs on proliferation of bone marrow progenitors could be reversed by tamoxifen, an estrogen antagonist abolishing estrogen receptor-mediated *TERT* expression, thereby underscoring the involvement of *TERT* in AD mechanism of action. In conclusion, no significant differences in the ability to functionally enhance telomerase activity could be observed for the three AD studied in vitro. Physicians therefore might choose treatment based on patients’ individual co-morbidities, e.g., pre-existing liver disease and expected side-effects.

## 1. Introduction

Telomeres are important DNA repeat sequences located at the end of the eukaryotic chromosomes. They act as a protective cap to avoid the exposure of single stranded DNA to the cellular DNA damage response machinery [1]. The synthesis of telomeres is catalyzed by the enzyme telomerase, which is particularly active during development [2]. In most somatic cells including most hematopoietic cells [3,4] and across many species [5], telomeres shorten with each cell division in vitro and in vivo until a critical short telomere length is reached. The DNA repair complexes recognize critically short telomeres and trigger cellular senescence and/or apoptosis [6]. Hematopoietic stem cell (HSC) transplantation [7,8,9], as well as acquired bone marrow failure syndromes [10,11] are characterized by increased HSC turnover and consequently, are associated with accelerated telomere shortening (reviewed by Brümmendorf and Babalanov et al.) [12]. Even more, genetic disorders caused by an altered functional capacity of telomerase such as classical dyskeratosis congenita (DKC) represent paradigmatic diseases to study the functional consequence of impaired telomere maintenance [13] DKC is a rare disease characterized by bone marrow failure (BMF) and a triad of mucocutaneous signs such as skin pigmentation, nail dystrophy and oral premalignant leukoplakia [14]. DKC has a highly variable clinical presentation, particularly regarding the severity and the involvement of non-hematopoietic organ systems, eventually leading to interstitial lung fibrosis, gut involvement or idiopathic liver cirrhosis [13] as well as a dramatically increased rate of secondary solid and/or hematopoietic malignancies. The clinical symptoms of DKC patients are caused by prematurely shortened telomeres due to mutations in genes coding either for components of the telomerase complex or in elements involved in telomere replication or stabilization [15]. The most common mutations affect the telomerase enzyme complex itself with defects in the telomerase reverse transcriptase subunit (*TERT*), or the telomerase RNA component (*TERC*). However, various other mutations affecting helicases, namely poly (A)-specific ribonuclease (*PARN*) or regulator of telomere elongation helicase 1 (*RTEL1*), or the ribosomal core component H/ACA ribonucleoprotein complex subunit DKC1 (*DKC1*) are found [14]. The two currently established therapeutic options for patients with DKC and bone marrow failure (apart from symptomatic treatment) are allogenic hematopoietic stem cell transplantation (HSCT) or treatment with androgens derivatives (ADs) [16]. Unfortunately, due to the substantial treatment-associated morbidity and mortality, HSCT is frequently not an attractive option for patients with DKC. In addition, patients often either lack a suitable HSC donor or a potential sibling donor albeit yet still asymptomatic is affected by the same inherited mutation (which is why telomere screening is recommended for family members). Even more importantly, severe affection of non-hematopoietic organs, such as the lungs or liver, often preclude DKC patients from using HSCT as treatment due to the risk of excess toxicity and morbidity [16].

Therapies with ADs, such as nandrolone, oxymetholone or danazol have been employed for the treatment of BMF syndromes, especially aplastic anemia, since the 1960s with variable results [17]. For DKC patients, recent studies showed promising results with improvement of peripheral blood counts and, at least in some patients, elongation of prematurely shortened telomeres [18,19,20]. These studies showed that treatment with AD was mediated by up-regulation of *TERT* via the stimulation of the intracellular estrogen receptor and, as a consequence, enhancement of the enzymatic activity of telomerase [18,21]. Thus, haploinsufficiency of telomerase components in most patients with DKC can be counterbalanced by increasing telomerase activity via stimulation of *TERT* expression. Recently and in line with these studies [18,19,20], we could show that long-term treatment with danazol and oxymetholone improved blood counts and elongated telomere length in patients with DKC and mutations in *TERT* and *TERC* genes [22]. However, so far, prospective clinical studies comparing different AD are missing, mostly due to the rareness of this disease and the clinical diversity of the affected patients. Limited data from registries point to potential differences in responses to oxymetholone and danazol in patients with distinct entities of inherited BMF [23]. However, results from in vitro testing regarding the efficacy of the different AD compounds in individual patients has not been reported to date.

Consequently, we tested the differential activity of the three most frequently used ADs, namely danazol, oxymetholone and nandrolone in four patients with DKC in vitro.

## 2. Results

### 2.1. DN, OX and ND Exert a Comparable Effect on the Proliferation Rate of Patient-Derived MNC

**Figure 1:**
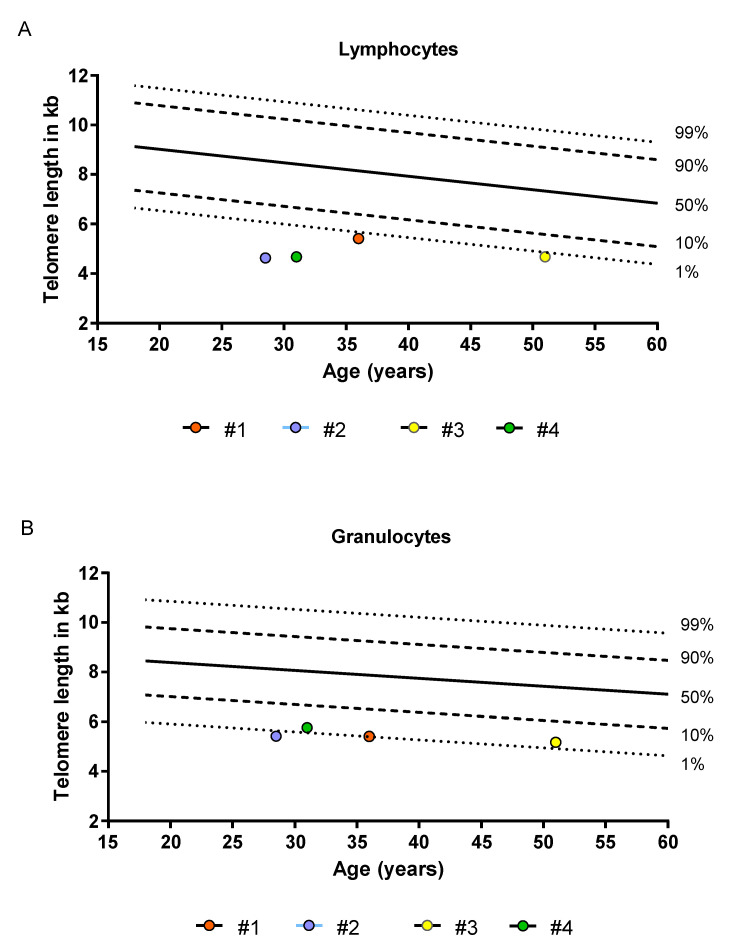
Telomere length of four patients analyzed in comparison to telomere length distributions derived from healthy individuals. Telomere length was assessed with flow-FISH as previously described [24,25,26]. (**A**) Lymphocyte telomere length (**B**) Granulocyte telomere length. The dashed and dotted lines represent the respective percentiles (1%, 10%, 50%, 90% and 99%) of healthy individuals.

MNCs from the peripheral blood (PBMCs) of three DKC patients (Patient #1, #2 and #3, Table 1, Figure 1A,B) and one healthy donor were stimulated with phytohemaglutinin (PHA) and interleukin-2 (IL-2) in order to expand the T-lymphocyte fraction.

**Figure 2:**
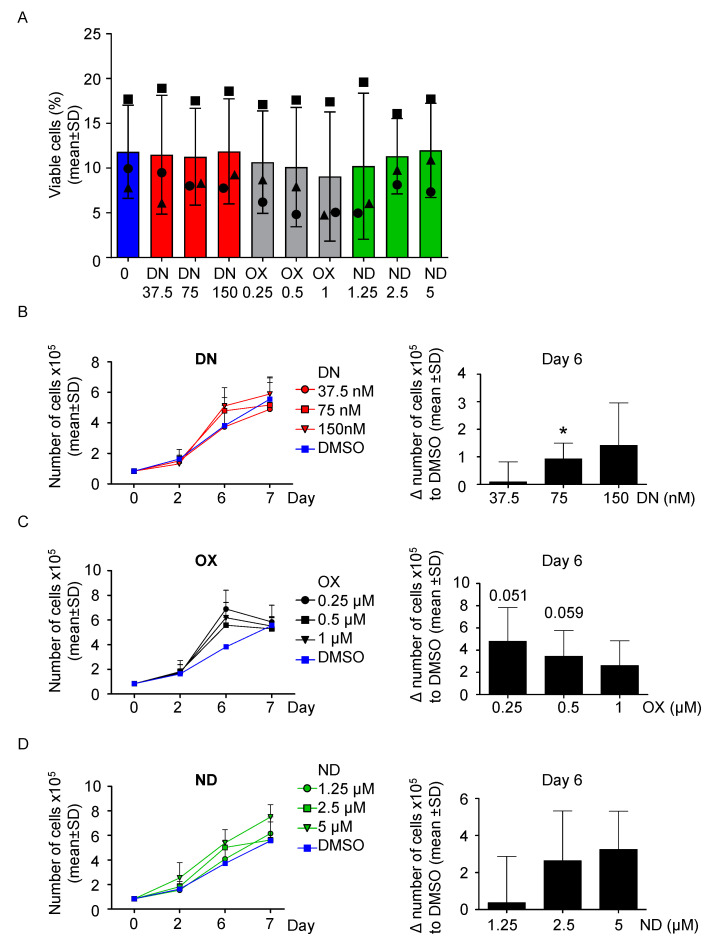
All ADs increase the number of cells DKC-derived PBMCs in vitro in a comparable fashion. (**A**) PBMC were treated for up to 9 days with three increasing concentrations of danazol (37.5 nM, 75 nM and 150 nM), oxymetholone (OX, 0.25 µM, 0.5 µM and 1 µM), as well as nandrolone (ND, 1.25 µM, 2.5 µM and 5 µM). At day 9, viability was determined by PI staining and analysis via fluorescence-activated cell sorting (FACS). Patient #1 is represented with a circle, Patient #2 with a triangle and patient #3 with a square. (**B**) PBMC were treated with danazol, as described in panel A. The number of cells was assessed versus the vehicle control (DMSO) at day 0, 2, 6, and 7. On the left, growth curves for each condition are shown. On the right, the difference between each treated condition and DMSO was calculated and shown into a bar graph. One sample *t*-test was used to determine statistical significance. (**C**) The experiment of panel B was also performed using the three concentrations of oxymetholone (OX, 0.25 µM, 0.5 µM and 1 µM). (**D**) The experiment of panel B was also performed using the three concentrations of nandrolone (ND, 1.25 µM, 2.5 µM and 5 µM). * *p* < 0.05.

First, to evaluate the effect of the ADs on healthy cells, 100,000 PBMC/mL derived from the healthy donor were cultured with three increasing concentrations of each ADs, namely danazol (DN), oxymetholone (OX) and nandrolone (ND) up to 9 days, based on previous publications [21,27,28] in order to compare their effect on cell proliferation. No relevant effect on viability from all androgens was detected (Figure S1A–C). Interestingly, cells were still viable after nine days of culture in all conditions tested (Figure S1D). Next, PBMC from the DKC patients were cultured following the same conditions described above. After 7 days of culture, the cells in each condition ceased to show signs of proliferative activity and most cells underwent cell death at day 9 (Figure 2A). As shown in Figure 2, all compounds used caused a substantial temporary increase in the cell number (Figure 2B–D), with DN 75 nM being most effective after 6 days of treatment (*p* < 0.05, Figure 2B). OX was also found most effective after 6 days of culture, with 0.25 and 0.5 µM treatments with approaching the threshold for statistical significance (*p* = 0.051, *p* = 0.059 respectively, Figure 2C). Similar findings were observed for ND, but at its lowest dose tested (1.25 µM) it was not able to increase the proliferation rate after 6 days of treatment (Figure 2D). In summary, AD-treated cells reached the maximal proliferation rate earlier before undergoing cell death, explaining the only temporarily improvement compared to DMSO treatment. Of note, increasing the concentration of DN and ND caused a proportional improvement of the proliferation rate, whereas OX did not (Figure 2B–D).

### 2.2. DN and OX Increase TERT Expression in Both PBMC and BM-MNC

**Figure 3:**
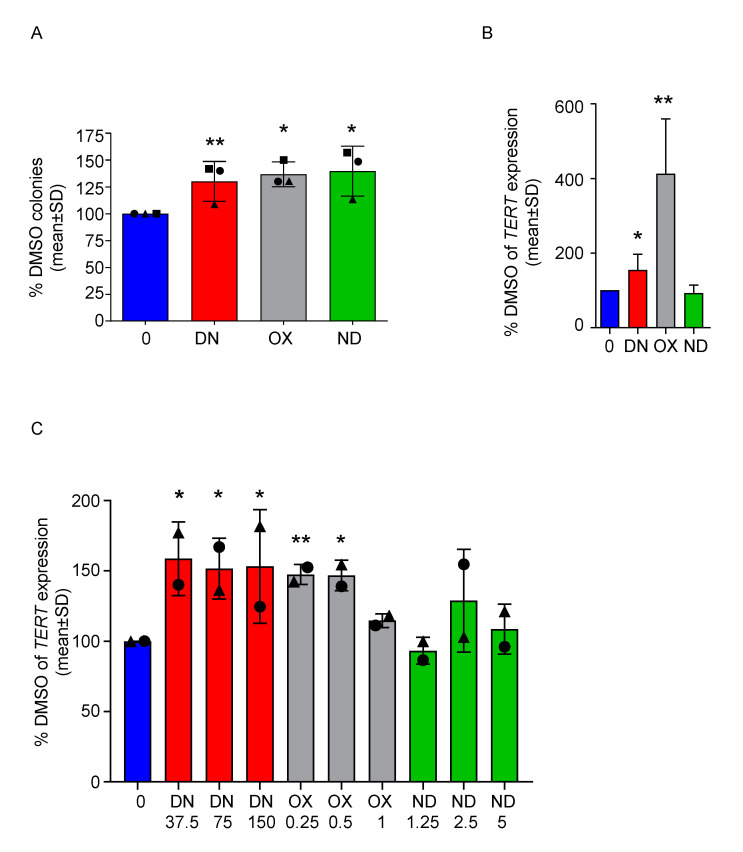
All ADs improve colony-forming capacity of DKC-derived BM-MNCs and danazol and oxymetholone increase the expression of *TERT* in PBMC of DKC patients. (**A**) BM-MNCs were cultured in a semisolid medium in presence of the highest doses of ADs depicted in the previous figure (DN 150 nM, OX 1 µM and ND 5 µM) versus DMSO, in order to perform a CFU assay. The number of colonies was assessed after 7 days and normalized towards the control value. One sample *t*-test was used to determine statistical significance. This experiment was performed with three different patient samples in independent experiments and results are showing their mean values ± SD. Patient #1 is represented with a circle, Patient #2 with a triangle and patient #3 with a square. (**B**) *TERT* mRNA levels in colonies obtained from the CFU assay of patient #1 were determined. Values were normalized towards DMSO values and one sample *t*-test was used to determine statistical significance. (**C**) *TERT* mRNA levels were determined in PBMC treated with ADs at different concentrations for 7 days (DN 37.5, 75 and 150 nM; OX 0.25, 0.5 and 1 µM; ND 1.25, 2.5 and 5 µM ). *TERT* levels were normalized towards DMSO values and one sample *t*-test was used to determine statistical significance. This experiment was performed with two different patient samples (patient #1, circle and #2, triangle) in independent experiments and results are showing their mean values ± SD. * *p* < 0.05, ** *p* < 0.01.

Next, we performed a colony forming unit assay (CFU) with MNCs isolated from the bone marrow (BM-MNCs) of the same patients after 7 days of culture. Based on the results from PBMC, we used the highest concentration for each CFU. We could observe a significant increase in the number of colonies in all conditions compared to DMSO (*p* < 0.0068 for DN 150 nM, *p* < 0.045 for OX 1 µM and *p* < 0.03 for ND 5µM, Figure 3A). Moreover, since androgens are known to stimulate telomerase gene expression [21], we tested whether the three ADs tested could lead to comparable *TERT* mRNA up-regulation. For patient #1, a sufficient number of colonies was harvested from each condition illustrated above, allowing the measurement of *TERT* expression on BM-derived cells treated for 7 days with ADs in vitro. Both DN and OX caused a significant increase of TERT (*p* < 0.046 and *p* < 0.0034 respectively, Figure 3B). For patients #1 and #2, *TERT* expression was also measured in PBMC treated for 7 days with ADs (Figure 3C), showing a significant up-regulation upon treatment with any concentration of DN (*p* < 0.012 for DN 37.5 nM, *p* < 0.018 for DN 75 nM, *p* < 0.028 for DN 150 nM) and two out of three concentrations of OX (*p* < 0.0012 for OX 0.25 µM, *p* < 0.015 for OX 0.5 µM). Similar results were also obtained a healthy donor (Figure S1E), where nearly all concentration of DN and OX tested caused a slight, but significant up-regulation of TERT levels (*p* < 0.034 for DN 37.5 nM, *p* < 0.034 for DN 150 nM, *p* < 0.017 for OX 0.25 µM, *p* < 0.011 for OX 0.5 µM and *p* < 0.03 for OX 1 µM) similar to previous reports [21]. *TERC* levels were either very low or undetectable in both DKC patients analyzed. In the healthy donor derived PBMCs, we could not observe a relevant modulation of *TERC* expression (Figure S2).

### 2.3. Danazol, Oxymetholone and Nandrolone Cause Telomere Elongation of BM-MNC and the Improved Proliferation Is Abolished by the Addition of Tamoxifen

**Figure 4:**
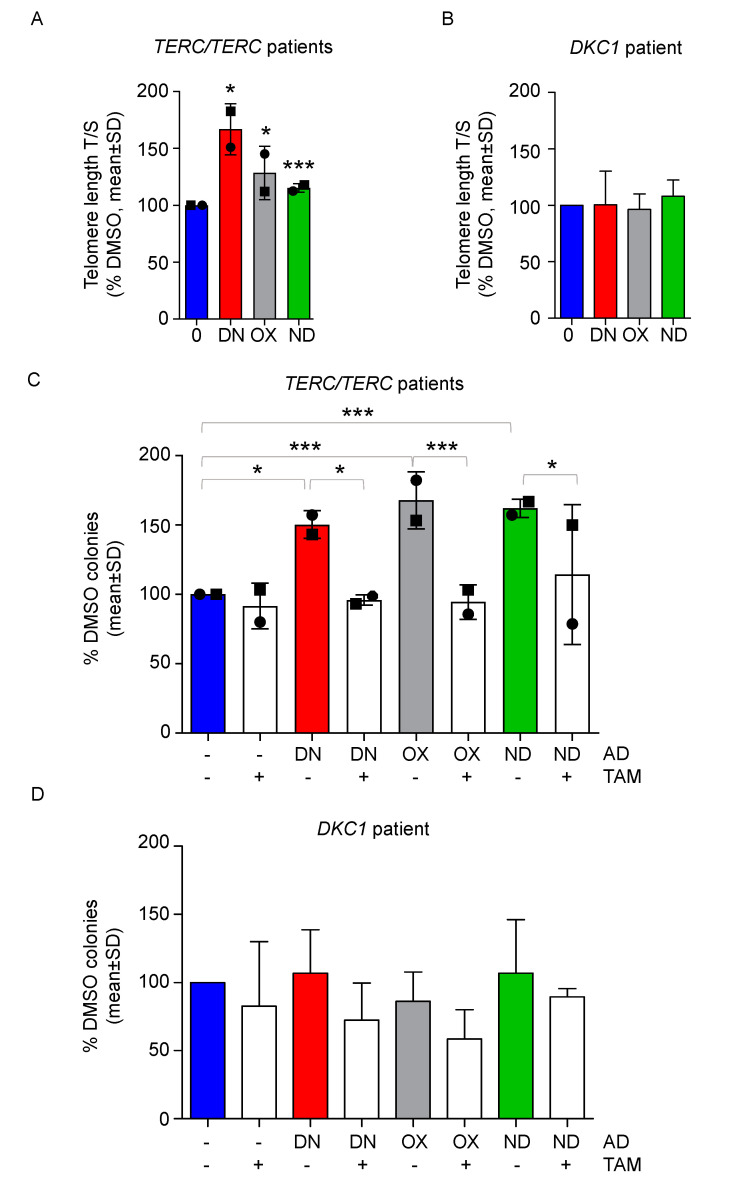
All androgens stimulate telomere elongation and tamoxifen reverts the positive effects of ADs in DKC-derived BM-MNCs with *TERT/TERC* mutations. (**A**) DKC-derived BM-MNCs from patients #1 and #3 (represented by a circle and a square, respectively), were treated for 7 days with vehicle, danazol 150 nM (DN), oxymetholone 1 µM (OX) and nandrolone 5 µM (ND). Next, telomere length was measured via qPCR. One sample *t*-test was used to determine statistical significance. (**B**) The same experiment was repeated for patient #4 and telomere length is shown. (**C**) CFU assay was performed with BM-MNC treated with the following agents: danazol 150 nM (DN), oxymetholone 1 µM (OX), nandrolone 5 µM (ND), tamoxifen 1µM (TAM) and selected combinations, such as DN + TAM, OX + TAM, ND + TAM versus DMSO. The number of colonies was assessed after 7 days and normalized towards the control value. One-way ANOVA was used to determine statistical significance. (**D**) The same experimental layout of panel C was employed with BM-MNC from patient #4. Mean values ± SD are shown for all experiments. * *p* < 0.05, *** *p* < 0.001.

Treatment with ADs is able to stimulate telomere elongation in patients with *TERC* or *TERT* mutations, as observed in our work [22] and in previous studies [18,19,20]. We therefore tested whether a relatively short treatment with ADs could cause an increase in telomere length in vitro in BM-MNCs. We could show a slight, but significant telomere elongation by DN, OX, and ND after seven days via quantitative real-time PCR (*p* < 0.013 for DN, *p* < 0.011 for OX and *p* < 0.0037 for ND, Figure 4A). On the other hand, in a patient with DKC1 mutation (patient #4) no significant telomere elongation was observed (Figure 4B).

Finally, we wanted to address whether the observed effects of improved proliferation are caused by the observed increased *TERT* expression and therefore by increased telomerase activity. We used tamoxifen (TAM), an estrogen antagonist able to abolish the estrogen receptor-mediated effects of the ADs. BM-MNCs from patients #1 and #3 were treated with the highest dosages of ADs in combination with TAM and after 7 days of culture, the effect on colony forming capacity was assessed, showing that TAM was able to significantly impair the positive effects on colony growth of all androgens tested (Figure 4C). BM-MNCs from patient #4 did not show either an increased clonogenic potential upon treatment with DN, OX, or ND or a significant decrease of colonies in combination with TAM (Figure 4D).

## 3. Discussion

Based on data obtained first from individual case reports [20] as well as eventually from prospective clinical trials [18], patients with DKC have increasingly been treated with ADs for the last years. However, no comparable data on the differential efficacy of the different ADs used have been published to date. In the current study, we present a functional in vitro comparison of the clinically used ADs. Interestingly, all compounds were found equally effective in improving the proliferation rate of both PBMC and BM progenitor cells from patients with two different DKC genotypes characterized by *TERC* or *TERT* mutations. Concerning their ability to enhance *TERT* expression, we could observe a significant up-regulation of *TERT* mRNA in *TERT/TERC* mutated DKC patients with DN and OX independent of the dosage applied, whereas ND was found to have only negligible effects on *TERT* expression here. However, all ADs caused a significant elongation of telomeres and improved proliferation of affected cells. Focusing on the mechanism, TAM was able to significantly revert the augmented colony-forming capacity of all ADs. TAM blocks the estrogen receptor, which is shown to be the main pathway of *TERT* activation in response to ADs [21]. This data strongly suggests that telomerase activation stimulated by the androgens is directly involved in the pro-proliferative effects on bone marrow cells, since its inhibition caused the androgens tested to lose their beneficial effects. However, we cannot rule out other potential mechanisms that may play a role in the effects observed. ADs are, e.g., able to activate the erythropoietin receptor in progenitor cells [29]. Of note, all effects including telomere elongation were observed in a relatively short time, in line with previous studies reporting clinically relevant response to AD treatment within the first three months in most cases [22,23].

Interestingly, the BM-MNC derived from the DKC patient carrying a *DKC1* mutation did not respond to any androgen treatment: the clonogenic potential was not augmented, as well as telomere length remain comparable in all conditions. Based on these results, we argue that androgens might be truly beneficial only in patients with a haploinsufficiency of the telomerase complex, whereas patients carrying mutations that impair the telomere maintenance with other mechanisms might be irresponsive to this therapy. However, definitive conclusions on the clinical efficacy of DN, OX, and ND in patients with other DKC-related mutations requires prospective collaborations of multiple centers.

For patients with *TERC/TERT* mutations, we speculate that all three drugs might be equally effective for the treatment of DKC, although ND failed to increase *TERT* expression at the specific time point analyzed. In fact, their ability to stimulate proliferation and telomere elongation seems to be comparable and the choice of the right compound might therefore be mostly guided by the patients’ comorbidities or with the aim to avoid specific side effects. OX is of limited benefit in women affected by DKC because of its high degree of virilization [30]. DN, although it has a toxicity profile that has yet to be completely determined [18,31], might cause an attenuated degree of masculinization and might be more appropriate for the treatment of women. Moreover, liver toxicity appears to be more common with OX [16]. Consequently, this compound might be avoided in favor of DN or ND in patients with pre-existent liver damage. On the other hand, OX could be indicated for patients with co-morbidities such as wasting disease, considering that it is known to cause weight and muscle gain in patients with other diseases [32].

Another important aspect that emerged from our study is that low-dose concentration of ADs showed relevant improvement of cell proliferation rate and similar *TERT* expression as higher dose AD. Of note, the dosage of oxymetholone reported in literature varies from 0.5–2 mg/kg/day [33]. Nandrolone is instead proposed to a dosage of 5 mg/kg every 14 days for 24 months [34]. For danazol, plasma levels in patients under treatment with 50, 100–200 or 400–800 mg/day danazol have been published and correspond to the concentrations used in our experiments [35]. Especially for DN, the most frequent used AD, our data support the rationale of a recent clinical trial comparing the efficacy of lower DN dosage with the standard dose (ClinicalTrials.gov Identifier: NCT03312400). Such a strategy could further reduce the onset of adverse effects or allow medication with additional drugs such as nintedanib for co-existing lung fibrosis without loss of efficacy. Without doubt, results of clinical trials addressing such issues are clearly needed to better address this concern.

A major challenge in this study was to come up with the collection of a homogeneous and representative patient cohort, given the rarity of the disease. We focused our investigation on patients with either *TERT* or *TERC* mutations since ADs are expected to increase telomerase activity in patients with mutation-related haploinsufficiency of such genes via increasing *TERT* expression and activity [22]. It remains speculative whether our observed effects can be achieved in patients whose telomerase complex is intact and have an indirect impairment of telomerase activity due to loss of function of other telomere biology parameters.

In conclusion, the choice of the most appropriate androgen derivative to treat DKC could be made on the basis of the side effects of each compound and possible lower doses of AD could be sufficiently effective given the comparable functional ability of AD to enhance telomerase activity in vitro.

## 4. Patients and Methods

### 4.1. Patients

Four patients enrolled in the Aachen Telomeropathy Registry were analyzed as part of this analysis. All samples were taken after written consent and according to approval by the local ethics committee (EK206/09, 5 January 2010, RWTH Aachen University). All patients had molecularly confirmed DKC (two with mutations in TERC, one in TERT and one in DKC1). TL assessments of peripheral blood granulocytes and lymphocytes were carried out by Flow-FISH as described previously [24,25]. The flow-FISH results were obtained in telomere fluorescence units (TFU) and translated in kb equivalents. All patient showed lymphocyte TL below the 1% percentile of a normal control cohort (Figure 1). Acquisition of peripheral blood and bone marrow biopsy were carried out on the same day. A summary of the major patient characteristics is found in Table 1.

### 4.2. Drug Studies

Danazol (DN), oxymetholone (OX), nandrolone (ND) and tamoxifen (TAM) were purchased from Sigma Aldrich (St. Louis, MO, USA) dissolved in DMSO at a concentration of 10 mM and stored at −20 °C for further experiments. Patient-derived cells were treated with the mentioned agents for specific time-point as stated in the according experiments. DMSO was used as vehicle control at a concentration of 0.05%. The concentration used were extrapolated and adapted from three previous publications [21,27,28]. For danazol, the concentrations (37.5, 75, 150 nM) used in our in vitro test recapitulate the plasma levels of patients treated for 15 days with 50, 100–200, or 400–800 mg/day danazol, respectively [35].

### 4.3. In Vitro Culture of Peripheral Blood Mononuclear Cells (PBMC)

Mononuclear cells (MNC) were isolated by gradient centrifugation with Ficoll paque (GE healthcare, Chicago, IL, USA). Next, cells were cultured in Roswell Park Memorial Institute medium (RPMI-1640, Invitrogen, Carlsbad, CA, USA) with GlutaMAX containing 10% fetal bovine serum (PAN-biotech, Aidenbach, Germany), 100 IU/mL penicillin, 100 μg/mL streptomycin (Invitrogen, Carlsbad, CA, USA) and in presence of phytohemagglutinin (5 μg/mL; Sigma-Aldrich, St. Louis, MO, USA) and interleukin-2 (40 IU/milliliter; PeproTech Inc, Rocky Hill, NJ, USA) to stimulate the expansion of T-lymphocytes. Cell were cultures at 37 °C in a humidified incubator with 5% CO_2_ for up to 7 days. Viability was determined with methylene blue exclusion staining at specific time-points, stated in the according experiments. In Figure 2A and Figure S1D, PBMC after 9 days of culture were resuspended in PBS with PI (0.2 μg/mL, BD Biosciences, San Jose, CA, USA ), used as a dead cells marker and were analysed by FACS (BD Accuri C6, BD Biosciences, San Jose, CA, USA).

### 4.4. Colony Forming Unit

Bone marrow samples were subjected to gradient centrifugation with Ficoll paque (GE healthcare, Chicago, IL, USA) to isolate and culture MNC for 7 days in a semisolid medium containing 80% methylcellulose (Stem Cell Technologies, Cologne, Germany), 20% Iscove’s Modified Dulbecco’s Medium (IMDM, Thermo Fisher, Waltham, MA, USA), 10−4 M 2-mercaptoethanol, 2 mM l-glutamine and supplemented with 50 ng/mL rhSCF, 10 ng/mL rhIL-3, 10 ng/mL rhGM-CSF, 3 U/mL rhEPO (all Immunotools, Friesoythe, Germany) and 0.5% ciprofloxacin. 120.000 cells were plated in triplicates in 35 mm cell culture dishes (with grid, Thermo Fisher, Waltham, MA, USA) and incubated at 37 °C for seven days. Colony forming ability was evaluated by colony number quantification using an inverted light microscope. Ten colonies per condition were picked from the dishes after 7 days of culture and RNA or DNA were isolated for further experiments.

### 4.5. RNA Isolation and Quantitative Real-Time PCR (qPCR)

Total RNA from MNCs was extracted using miRNeasy Mini Kit (Qiagen, Hilden, Germany). cDNA was generated using random hexamers and the M-MLV Reverse Transcriptase (both Invitrogen, Carlsbad, CA, USA). Quantitative real-time PCR (qPCR) was performed with the SYBRGreen mix (Invitrogen, Carlsbad, CA, USA) and the ABI7500fast real-time PCR system (Applied Biosystems, Foster city, CA, USA) according to standard PCR conditions. Primers used for qPCR are the following: TERT-F: CGG AAG AGT GTC TGG AGC AA; TERT-R: GGA TGA AGC GGA GTC TGG A; TERC-F: TCC ACC GTT CAT TCT AGA GCA; TERC-R: ACT CGC TCC GTT CCT CTT C; MT-ATP6-F: CGT ACG CCT AAC CGC TAA CA; MT-ATP6-R: AGG CGA CAG CGA TTT CTA GG. MT-ATP6 was used as a housekeeping gene.

### 4.6. DNA Isolation and Telomere Length Analysis by qPCR

DNA from BM-MNC was extracted for using the DNeasy Blood & Tissue Kit (Qiagen, Hilden, Germany) according to the manufacturer’s instructions. For the telomere length (TL) analysis by qPCR, 1.4 ng of genomic DNA was used per reaction. TL qPCR was performed using the Absolute Human Telomere Length Quantification qPCR Assay Kit (ScienCell, Carlsbad, CA, USA) and FastStart Essential DNA Green Master (Roche, Basel, Switzerland). Leukocytes from healthy subjects (*n* = 104) were used for age adaptation of TL, which is given in T/S ratios [26]. A T/S ratio is calculated by dividing the number of copies of the telomere template (T) by the single copy reference (SCR) template (S) which is an amplified 100 bp-long region on human chromosome 17. The TL qPCR was performed according to the manufacturer’ instructions.

### 4.7. Statistical Analysis

Statistical analysis was performed with GraphPad Prism (GraphPad Software version 8.1.0, La Jolla, CA, USA). One-way ANOVA was used for multiple comparison analysis, employing the Bonferroni multiple comparison post-test. The one sample *t*-test was applied for single comparisons of normalized data towards the control condition. *p*-values < 0.05 *, <0.01 ** and <0.001 *** were considered as statistically significant.

## Figures and Tables

**Table 1:** Clinical characteristics of the three analyzed patients. M: male; F: female. The age is given in years.

ID	Sex	Age	Clinical Symptoms	Muco-Cutaneous Signs	Family History	Genotype	Leuko-Cytes/nL	Hb g/dL	Thrombocytes/nL
**#1**	M	36	Cytopenia, early hair greying	No signs	No	TERC [n.73G>A]	2.8	13.2	38
**#2**	F	29	Cytopenia, liver fibrosis, early hair greying	Nail dystrophy	Brother with leukemia, Father with head and neck cancer	TERC [n.128A>G]	2.9	10.6	19
**#3**	M	51	Cytopenia, liver fibrosis, lung fibrosis, early hair greying	Nail dystropy	Three siblings died of lung fibrosis	TERT [c.2147C>T] p.Ala716Val	3.7	12.9	56
**#4**	M	31	Cytopenia, lung fibrosis, liver fibrosis, early hair greying	Nail dystrophy, leukoplakia	Brother died of lung fibrosis	DKC1 [c.146C>T] p.Thr49Met	6.0	11.0	98

## Supplementary Materials

Supplementary materials can be found at https://www.mdpi.com/1422-0067/21/19/7196/s1.
