# Supplementary material for: Comparable Effects of the Androgen Derivatives Danazol, Oxymetholone and Nandrolone on Telomerase Activity in Human Primary Hematopoietic Cells from Patients with Dyskeratosis Congenita
Source: Int J Mol Sci. 2020 Sep 29;21(19):7196. doi: 10.3390/ijms21197196 (PMC7584039; doi:10.3390/ijms21197196)
Supplement: Supplementary file 1 [file ijms-21-07196-s001.pdf]

Supplementary Figure 1

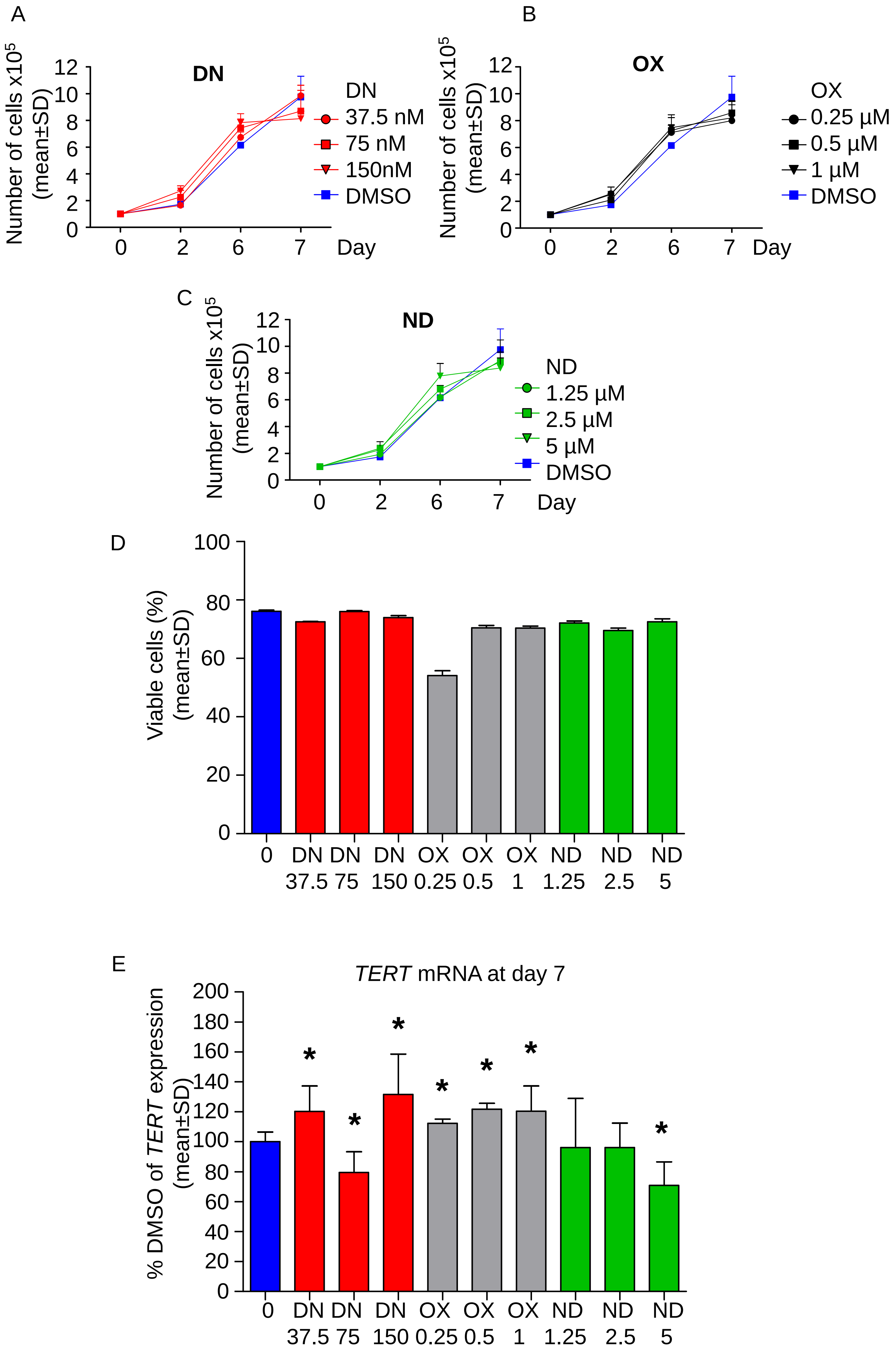

Supplementary Figure 1. Effect of danazol, oxymetholone and nadrolone on healthy donor-derived PBMCs. (**A**) PBMC were treated with danazol (DN) for up to 7 days with three increasing concentrations (37.5 nM, 75 nM and 150 nM) and the number of cells was assessed versus the vehicle control (DMSO) at day 0, 2, 6, and 7. Growth curves for each condition are shown. (**B**) The experiment of panel A was also performed using three concentrations of oxymetholone (OX, 0.25 µM, 0.5 µM and 1 µM, B), as well as nandrolone (ND, 1.25 µM, 2.5 µM and 5 µM) in panel **C**. (**D**) Viability of PBMC after 9 days of culture with the aforementioned concentrations of DN, OX and ND. Viability was determined by PI staining and analysis via fluorescence-activated cell sorting (FACS). One sample t-test was used to determine statistical significance. (**E**) *TERT* mRNA levels were determined in healthy donor-derived PBMCs treated with ADs at different concentrations for 7 days (DN 37.5, 75 and 150 nM; OX 0.25, 0.5 and 1 µM; ND 1.25, 2.5 and 5 µM). *TERT* levels were normalized towards DMSO values and one sample t-test was used to determine statistical significance.

Supplementary Figure 2

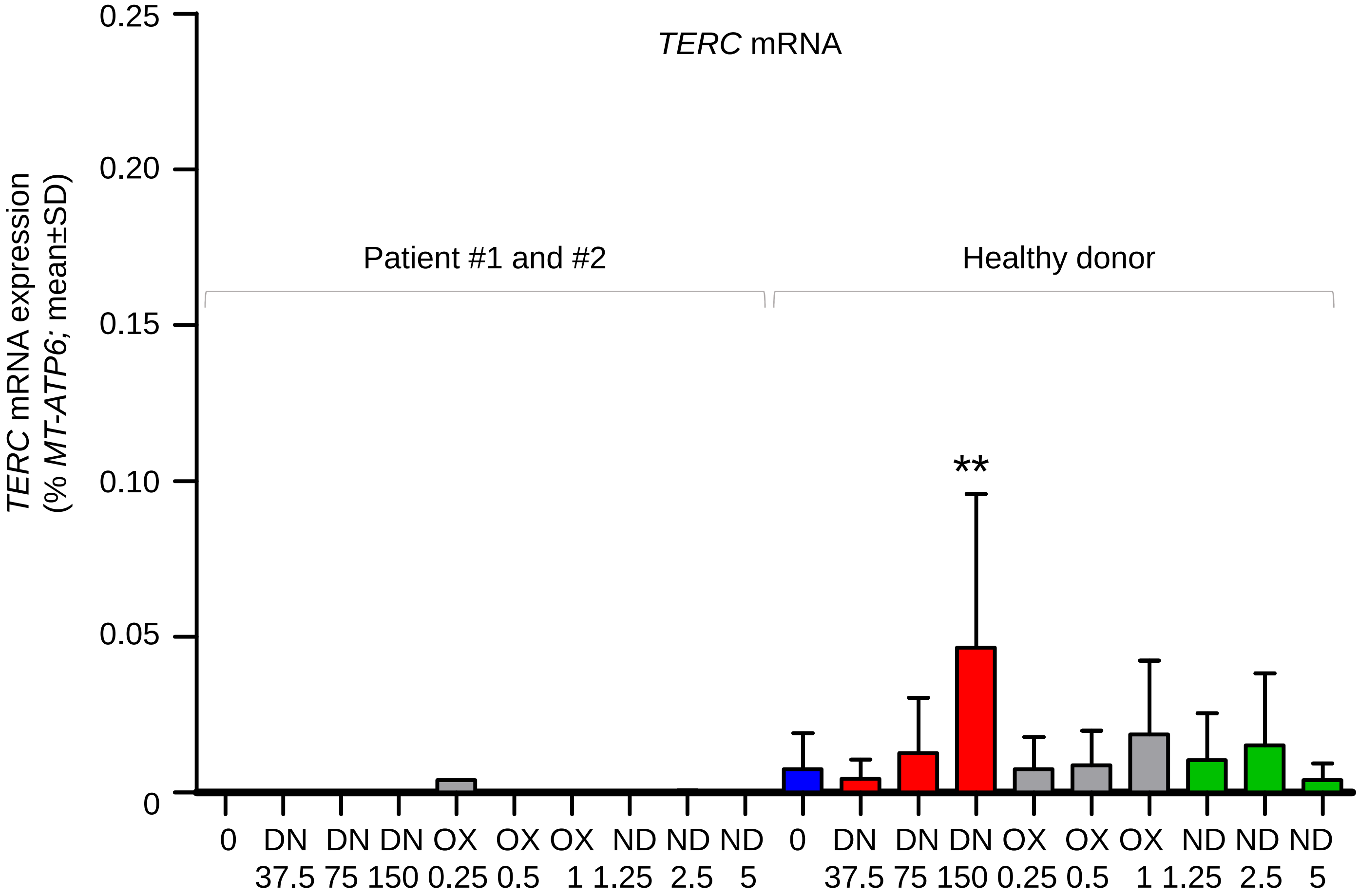

Supplementary Figure 2: Effect of danazol, oxymetholone and nadrolone on the expression of *TERC.* *TERC* mRNA levels were determined in DKC- (left) and healthy donor- derived (right) PBMCs treated with ADs at different concentrations for 7 days (DN 37.5, 75 and 150 nM; OX 0.25, 0.5 and 1 µM; ND 1.25, 2.5 and 5 µM). *TERC* levels were normalized towards the values of the housekeeping gene and one-way ANOVA was used to determine statistical significance.
